# Organometallic chemistry

**DOI:** 10.3762/bjoc.12.213

**Published:** 2016-10-19

**Authors:** Bernd F Straub, Rolf Gleiter, Claudia Meier, Lutz H Gade

**Affiliations:** 1 Organisch-Chemisches Institut, Universität Heidelberg, Im Neuenheimer Feld 270, D-69120 Heidelberg, Germany; 2 Department Chemie, Ludwig-Maximilians-Universität München, Butenandtstrasse 5–13 (Haus F), D-81377 München, Germany; 3 Anorganisch-Chemisches Institut, Universität Heidelberg, Im Neuenheimer Feld 270, D-69120 Heidelberg, Germany

This Thematic Series is dedicated to the memory of Professor Peter Hofmann (Figure 1) whose research work included many seminal contributions to the field of organometallic chemistry [1–3]. Organometallic chemistry has therefore been chosen as the topic of this Thematic Series of the *Beilstein Journal of Organic Chemistry*. Over a period of many decades, the chemistry of metal–carbon bonds has given rise to an ever-growing field of fundamental research and applied science, merging the reactivity of inorganic complexes with the structural diversity of organic compounds. The contributions in this Thematic Series reflect the broad impact of organometallic compounds in various fields of modern chemical research. These include the tailoring of new ancillary ligands, the investigation of more active and more selective homogeneous catalysts, catalytic transformations in the total synthesis of natural products, the theoretical and kinetic unravelling of reaction mechanisms, and the verification of rare coordination modes. These various facets underline the importance of organometallic chemistry in its own right, but also demonstrate the vital role that research in organometallic chemistry plays as a provider of invaluable tools for other chemistry sub-disciplines.

**Figure 1:**
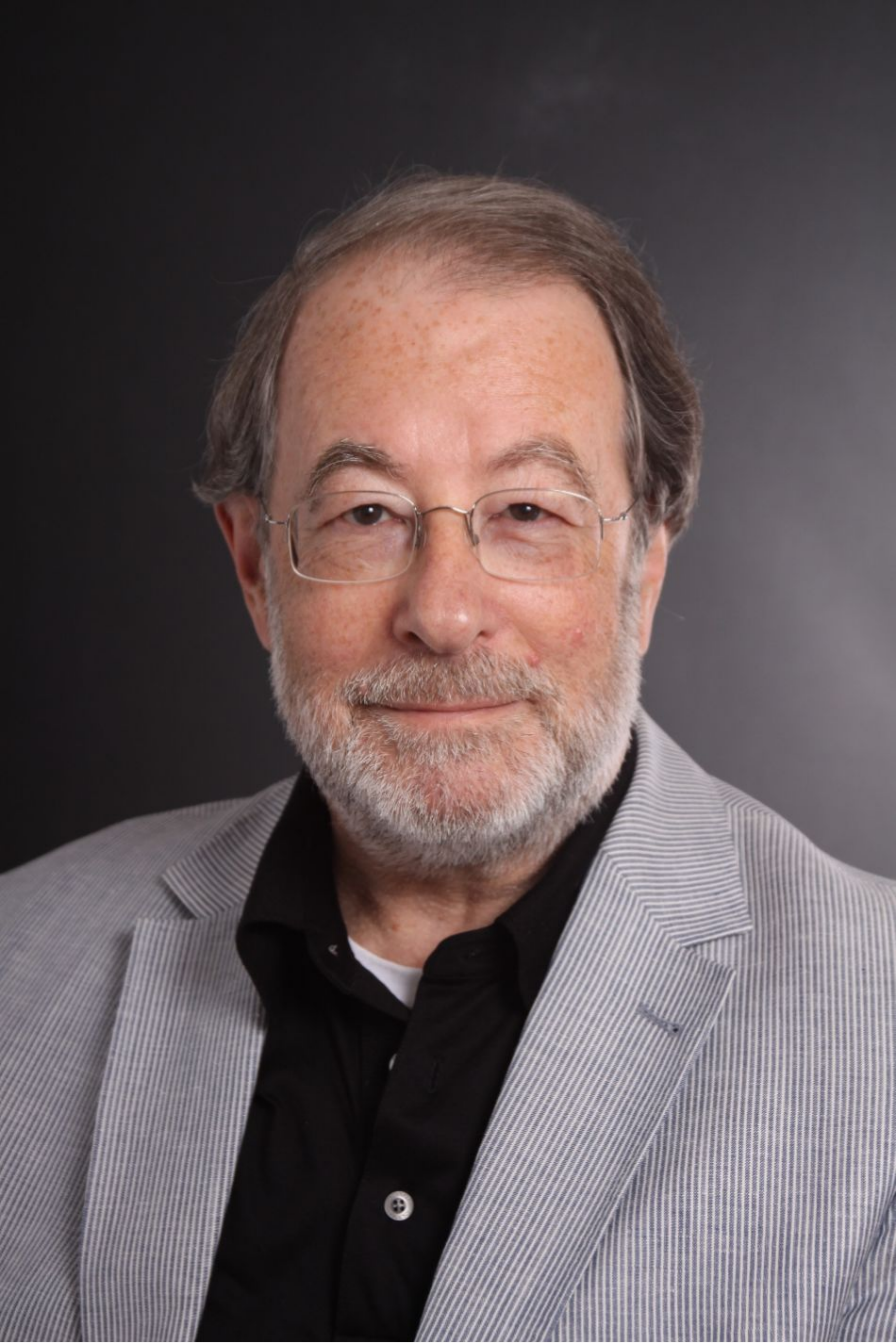
Peter Hofmann.

We thank the authors for their excellent contributions and are grateful for the commitment and the support of the Beilstein-Institut and its staff who made this Thematic Series possible.

## Peter Hofmann, a multifaceted organometallic chemist

Peter Hofmann died unexpectedly in Heidelberg on August 15, 2015, having retired from his chair position of organic chemistry at Heidelberg University just a few months before [4–155].

Peter Hofmann was born on January 12, 1947 in the Franconian city of Nuremberg, Germany, where he spent most of his youth. After the Abitur, his final secondary school examinations in 1966, he studied chemistry at the University of Erlangen-Nuremberg. In 1973, he received a doctorate with honors for his research on photochemical reactions of oxepin and thiepin derivatives in the group of Hans Hofmann [4–8,10,12,18]. Despite their identical family names, he and his doctoral supervisor were not related; Hof(f)mann is an unusually prevalent surname among distinguished German chemists.

In the time between his Ph.D. and postdoctoral research, he began his studies on vicinal triketones and their electronic structure [13,33,41].

Peter Hofmann joined the research group of Roald Hoffmann, the chemistry Nobel laureate of 1981, where he studied the electronic structure of organo-transition metal complexes by extended Hückel model calculations [14,17,20,28,39,49–50,54,57]. In 1978, he completed his habilitation in Erlangen, mentored by Paul von Ragué Schleyer, and was promoted to professor in 1980. Following a three-month visiting professorship in Berkeley in 1980, he was appointed to a visiting professorship in Munich in 1981 that was converted into a permanent academic position. Working with Ernst Otto Fischer, the chemistry Nobel laureate of 1973, he benefited from an ideal research environment that enabled him to compare his theoretical analyses and predictions with experimental data [34–38,40,42–43,45–60,62–63,65,74]. As an accomplished communicator of his research results, Peter Hofmann was a much sought-after visiting professor in the following years, which led to stints at the Universities of Bern, Ulm, TU and FU Berlin, Heidelberg, Rennes, Strasbourg, and Madison Wisconsin. In 1995, he declined the offer of a professorship from the Free University of Amsterdam and accepted a call to a chair position at Heidelberg University. He thus became one of the very few chemists with a history of research and teaching in theoretical, organic and inorganic chemistry. His research interests combined all three fields, with a focus on homogeneous catalysis with transition metal complexes. Bidentate donor ligands with a small bite angle such as bis(di-*tert*-butylphosphino)methane (dtbpm) were a unique feature of his work [61,67,70–72,80–81,84,86,93–94,97,100–101,103,107,124,128,148–149] that ultimately enabled C–Si activation of organosilanes and the C–C activation of oxiranes by coordinatively unsaturated platinum species [66,149]. In the mechanism of the Dötz reaction, he found that chromacyclobutene structures were unrealistic intermediates and instead proposed vinylcarbene complexes as much more stable isomers [64,68,88]. Other research projects of his group included the synthesis of ruthenium–carbene complexes with *cis*-dichloro ligands for olefin metathesis catalysis [86–87,90–91,98–100,108,132,138,147], the characterization of copper–carbene complexes as intermediates in the cyclopropanation of alkenes [102,106,113,119–120], as well as detailed studies into the mechanism of the hydroformylation of alkenes. An improved understanding of this industrially important process was achieved by a combination of ligand design for the rhodium catalysts, kinetic studies, and high-level quantum-chemical calculations [126,135,142,145–146,152,155].

In 2006, he initiated the foundation of the “Catalysis Research Laboratory” (CaRLa), a cooperation of Ludwighafen’s BASF and Heidelberg University, for which he acted as scientific head until October 2014. Postdocs from all over the world studied industrially important processes focusing on their fundamental principles [110,112,115,121,123,126–127]. The biennial “Heidelberg Forum of Molecular Catalysis” (HFMC), organized by Peter Hofmann until 2013, has been one of the most visible scientific events of Heidelberg’s chemical institutes.

Peter Hofmann was initiator, and until July 2009 chairman, of the SFB 623 “Molecular Catalysts: Structure and Functional Design”. As dean and vice-dean, he was actively involved in the affairs of the Heidelberg Faculty of Chemistry and Geosciences for six years. He received numerous awards and distinctions, including 2008 the Emil-Fischer medal of the GDCh and memberships in the Heidelberg Academy of Science and Humanities as well as the Academy of Science of North Rhine-Westphalia.

Peter Hofmann was an archetypical scientist and teacher, and in many respects a role model for colleagues and students. His scientific work revealed a high degree of intellectual creativity and deep chemical understanding, leading to modern and original research. He was reliable, honest and forthright in the interaction with his colleagues and students and enthusiastic when discussing any scientific problem. He preferred science over science politics and found it difficult to hide an aversion towards pomposity and concealed mediocrity, while being supportive towards less established junior colleagues.

We will remember Peter Hofmann as a tolerant, cooperative and amiable colleague.

Bernd F. Straub, Rolf Gleiter, Claudia Meier and Lutz H. Gade

Heidelberg, September 2016
